# A Novel Whole-Cell Mechanism for Long-Term Memory Enhancement

**DOI:** 10.1371/journal.pone.0068131

**Published:** 2013-07-11

**Authors:** Iris Reuveni, Drorit Saar, Edi Barkai

**Affiliations:** Departments of Neurobiology and Biology, University of Haifa, Haifa, Israel; Baylor College of Medicine, United States of America

## Abstract

Olfactory-discrimination learning was shown to induce a profound long-lasting enhancement in the strength of excitatory and inhibitory synapses of pyramidal neurons in the piriform cortex. Notably, such enhancement was mostly pronounced in a sub-group of neurons, entailing about a quarter of the cell population. Here we first show that the prominent enhancement in the subset of cells is due to a process in which all excitatory synapses doubled their strength and that this increase was mediated by a single process in which the AMPA channel conductance was doubled. Moreover, using a neuronal-network model, we show how such a multiplicative whole-cell synaptic strengthening in a sub-group of cells that form a memory pattern, sub-serves a profound selective enhancement of this memory. Network modeling further predicts that synaptic inhibition should be modified by complex learning in a manner that much resembles synaptic excitation. Indeed, in a subset of neurons all GABA_A_-receptors mediated inhibitory synapses also doubled their strength after learning. Like synaptic excitation, Synaptic inhibition is also enhanced by two-fold increase of the single channel conductance. These findings suggest that crucial learning induces a multiplicative increase in strength of all excitatory and inhibitory synapses in a subset of cells, and that such an increase can serve as a long-term whole-cell mechanism to profoundly enhance an existing Hebbian-type memory. This mechanism does not act as synaptic plasticity mechanism that underlies memory formation but rather enhances the response of already existing memory. This mechanism is cell-specific rather than synapse-specific; it modifies the channel conductance rather than the number of channels and thus has the potential to be readily induced and un-induced by whole-cell transduction mechanisms.

## Introduction

The increase in synaptic strength that mediates memory formation through Hebbian-type learning is traditionally thought to be synapse-specific, where mostly the synapses that connect a subset of active neurons are enhanced [1]. Taken together with the notion that learning involves both potentiation and depression of synaptic strength [2], the overall increase in excitatory synaptic strength into any particular cell should be relatively small. Indeed several studies which reason that Hebbian learning underlies the increase in synaptic strength demonstrate a small increase in the total synaptic strength following learning [3]–[8].

However, recently, a growing body of evidences demonstrates large overall increase in synaptic strength (>50%) following various training paradigms, in different brain structures [9]–[15]. Such enhanced synaptic transmission is transient, returning to baseline few days after training termination [9], [12]. The functionality of this increase is yet to be described; its magnitude and its transient nature do not agree with the principle of classical Hebbian learning.

We have previously shown that acquiring the skill to perform in a particularly difficult olfactory-discrimination task [15]–[20] results with a robust enhancement of excitatory as well as inhibitory synaptic connectivity to and within the piriform cortex that lasts for days after learning [15]–[18]. Recently we showed, using whole cell patch clamp recordings of miniature post synaptic currents (mPSCs) from pyramidal neurons, that olfactory discrimination learning-induced enhancement of synaptic transmission in cortical neurons is mediated by a robust increase of post synaptic modulation of AMPA receptor-dependent currents, and balanced by enhancement of post-synaptic GABA_A_ receptor-mediated currents. The synaptic enhancement was observed few days after the rats were last trained and thus indicates a long term induced synaptic modifications. Moreover, while an increase in excitatory and inhibitory mPSCs amplitude was evident in most of the recorded neurons, a subgroup that entailed a quarter of the cells showed an exceptionally great increase in the amplitude of spontaneous events [17]. In this sub group of neurons, most recorded synapses were strengthened after learning. While these results are in line with previous findings they are also incompatible with the expected from Hebbian learning.

The aim of the present study was to further describe quantitatively how synaptic weights are modified by complex learning and to explore the functional significance of such modulation to the cortical network activity, with the ultimate goal of describing the mechanism underlying long-term memory of elaborated performance capabilities. We show how long-lasting synaptic modifications are combined at different levels, from single synapses, through whole-cell modifications and to the network level, to enable the enhancement of high-skill memory.

## Results

### Synaptic enhancement is dominated by multiplication of AMPAR-mediated currents

We have shown recently [17] that learning induces a robust increase in the averaged mEPSC's amplitude following learning. In this study, the averaged mEPSC amplitude in neurons from trained rats was 64% higher compared to averaged value in neurons from naïve and pseudo-trained rats which did not differ (Averaged mEPSC amplitude 14.3±6.3 pA in neurons from trained rats, 8.4±3.3 pA in neurons from naïve rats, and 8.7±1.8 pA in neurons from pseudo-trained rats). As was shown previously [17] while the increase was evident in most recorded neurons, a sub-group of cells exhibited an exceptionally large increase in averaged events amplitude. Moreover it was shown that in this group of cells almost all synapses had increased their strength [17].

**Figure 1:**
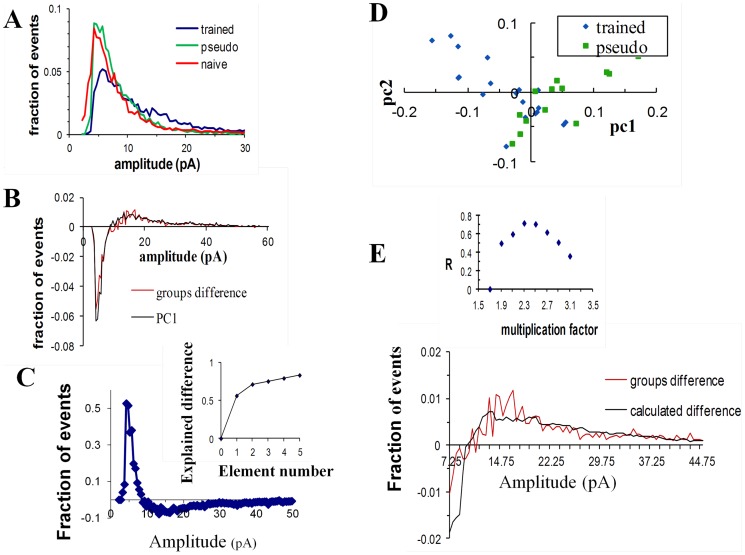
The learning-induced modulation of the excitatory unitary synaptic events amplitude is dominated by a single process in which the events amplitude was doubled. **A.** For each neuron an amplitude distribution curve was reconstructed from the mEPSC amplitudes and normalized (data taken from [17]). Averaged amplitude histograms, were calculated for all neurons from the three groups. Notably, a much more significant portion of events in the trained group are of high values. **B.** The curve calculated by subtracting the averaged pseudo amplitude distribution curve from the averaged trained amplitude distribution curve (red) match the first component calculated by PCA (black). The PCA component was scaled in the Y-axis, such that the best fit is achieved. We reasoned that such scaling is valid since this component is used by the PCA inside a linear combination. **C.** The first (major) component calculated by the PCA analysis. Inset: The first component accounts for 60% of the differences between amplitude distributions of all cells. **D.** For each cell the weight for the first component (X-axis) was drawn against its weight for the second component (Y-axis). Only the weights of the first component are significantly different between groups. **E.** For each multiplication factor a different curve that describes the difference between groups assuming a multiplication model was calculated. The calculated curve assuming multiplication factor of 2.2 (black) matched the curve that describes the main difference between groups (red). Inset: the correlation coefficient was calculated for each multiplication factor (for calculation of r only amplitudes >7pA were used, since at lower amplitudes, multiplication factors bigger than two requires unavailable data in amplitudes <3pA, see Methods).

To further explore the nature of synaptic modifications, normalized amplitude-distribution histogram of previously measured [17] miniature events was constructed for each neuron, and then averaged for each group. The difference between groups is apparent in figure 1A were the distribution curves of the neurons from the same group were averaged. While the amplitude distribution curves of the pseudo-trained and the naïve groups were similar, the amplitude distribution curve of the trained group was markedly different. After learning, the fraction of events with smaller amplitudes (around 8pA) was considerably decreased while the number of events with big amplitudes (>15pA) had increased which resulted with a slower decay of the amplitude distribution curve of the trained group.

Subtraction of the averaged amplitude distribution curve of the pseudo group from the one of the trained group yields the curve that describes the averaged difference between the pseudo and the trained groups (figure 1B). If there is a consistent difference between cells in the pseudo and the trained groups, then the difference between groups as described by this curve should dominate the within-group differences.

The largest difference between the distribution curves could be obtained by applying to the set of all curves a model-free analysis, principal component analysis (PCA, see Methods). This analysis calculates a number of arbitrary components that best portray the differences between all curves. The first principal component accounts for as much of the variability in the data as possible, and each succeeding component accounts for as much of the remaining variability as possible. In addition, for each distribution curve PCA assigns a set of weights, where each weight represents the relative power of its corresponding component in the distribution curve.

The first component (figure 1B) could explain more than 50% of the variance of all distribution curves; the second component could describe 15% of the variance while the third and higher components could explain less than 3% (figure 1C). Post-hoc examination of each group (figure 1D) revealed that the weights of the first component in cells from the trained group (−0.042±0.062, n = 22) was significantly different (P<0.0003) from the pseudo-trained group (0.057±0.079, n = 14). However the weights of the second and higher components were similar between groups (P<0.7, figure 1D). This led us to assume that the first component captures most of the inter-group differences.

If most of the variance in the data set results from the differences between groups, the inter-group variance should dominate the within-group variance for all amplitude. Following this, the curve that describes the differences between groups' averages should closely resemble the first PCA component. A set of simulations (table S1, table S2, and table S3) demonstrates that when the difference between groups is moderate (65% in the data; 75% in the simulations), the difference between group averages tends to closely resemble the first principal component if one process dominate the differences between groups. This can be explained as follows: If one process is dominant in creating the difference between groups, it will create a coherent difference between groups and thus enable the inter-group variance to dominate the within group variance for all amplitudes.

We found that the curve that describes the differences between groups averages closely resembles the first PCA component (R = 0.81, figure 1B), thus indicating that one process might underlie the difference between groups. Moreover we found that the within group variability can be mostly described by one linear combination of the second and the first component (figure S1). Together with the observation that the weights of the second component are similar between groups, this indicates that the first component is sufficient to describe most of the difference between distribution curves of the pseudo and trained groups, and thus further strengthening our hypothesis that one process dominates this difference.

We next aimed to reveal which process underlies the difference between the two groups by examining how the difference we had observed may be induced. Two different types of processes can underlie a robust post synaptic enhancement: an additive model in which the synaptic current is increased by a constant and a multiplicative model in which the synaptic current is multiplied by a constant. We showed that one process should dominate the difference between groups and that this constant should have roughly the same value for all synapses, and thus that the same calculation can be applied on all events. For each of the models we varied the constant and calculated fraction of synapses that have to be modulated in order to get the observed 64% difference in averaged event amplitude (see methods). Using these two parameters we could calculate a curve that describes the difference between groups, assuming this model. The difference between groups assuming an additive models did not match the observed experimental difference (R = 0) for any constant. For multiplicative models with multiplication factors between 2.3–2.5 the calculated curve match well with the experimental curve (figure 1E). Moreover, in neurons from trained rats, the increase in events amplitude was accompanied by a significant similar increase in their standard-deviation (the averaged SD in pseudo: 5.08±1.88; in trained: 8.18±4.39; P<0.02), further indicating that synaptic enhancement is obtained via a multiplicative, rather than an additive process. These results are in agreement with previous works which show a multiplicative increase of AMPAR-mediated conductance by a similar factor of 2.2–2.5 following LTP induction [21]–[23].

Further support for the observation that a two-fold multiplication process underlies the difference between groups is presented in figure S1. We showed that the curve describing the main differences between the different amplitude distribution curves in the trained group is a two-fold expansion (in the X-axis) of the curve describing the main differences within the pseudo group. This observation further supports our hypothesis that a single process in which the events amplitudes were multiplied by two underlies the differences between groups.

Thus, our analysis indicates that the major process which might underlie the difference between pseudo and trained group is a multiplication of the events amplitudes by a factor of 2.3–2.5.

### In a sub-group of neurons all synapses double their strength

**Figure 2:**
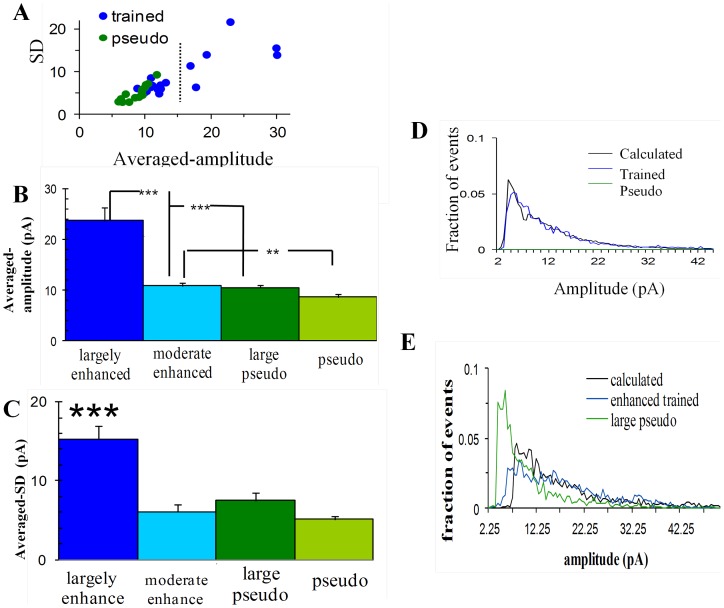
In a sub-group of cells from trained rats' amplitudes of all excitatory miniature events is doubled. **A.** Each cell was plotted as a function of its averaged event amplitude and standard deviation. Few cells from trained-group had exceptionally large averaged amplitudes and Standard-deviations. Using hierarchical clustering analysis the cells were divided to two groups (separated by the dotted line). **B.** The averaged amplitude of the greatly-enhanced-trained-group only is doubled to a value that is significantly higher than that observed for the three other represented sub-groups. Note that while the average amplitude of the moderately enhanced group is significantly lower than that of the greatly enhanced group, it is still higher that the averaged amplitude of the pseudo trained group. Values represent mean ± SE (**, p<0.01 ***, p<0.001). **C.** The standard deviation of the greatly-enhanced-trained-group is also doubled, compared to the other three sub-groups, which have all similar values. Values represent mean ± SE, (***, p<0.001). **D.** The distribution curve describing the trained neurons can be constructed from the events amplitudes of the pseudo-trained neurons. The expected curve (black) calculated from pseudo events (green) overlaps (r = 0.96) the trained distribution curve (blue). **E.** The distribution curve describing the greatly-enhanced trained neurons can be constructed from the events amplitudes of the large pseudo-trained neurons. The expected curve (black) calculated from events of the 4 biggest cells in the pseudo group (green) is similar to the averaged distribution curve of the greatly-enhanced-trained group (blue). (r = 0.73, both in D and in E only amplitudes >7pA were used, since at lower amplitudes, multiplication factors bigger than two requires unavailable data in amplitudes <3pA, see Methods).

Multiplication by a constant results in multiplication of the average and standard deviation by the same constant. Indeed the cells in the trained group exhibit an increase in standard-deviation that is similar to the increase in average (figure 2A). As was noted previously, not all cells exhibit a similar increase [17]. While the majority of the cells exhibited a small increase and had averaged amplitude values close to those in the pseudo group, a small group of cells exhibited a prominent increase both in amplitude and in standard deviation (figure 2A). Using hierarchical clustering analysis [24] we divided the neurons in the trained group to two distinct sub-groups that differ in their averaged amplitudes and standard deviations (figure 2A). The first sub-group, termed ***greatly-enhanced*** neurons, entails six cells (27% of the trained group) with very large means (22.7±5.9 pA) and large standard-deviations (13.8±4.9). Greatly-enhanced neurons are those in which the vast majority of synapses are enhanced after learning [17]. The second sub-population, termed ***moderately-enhanced*** neurons (all other 16 cells) had significantly (P<0.001) smaller means and standard-deviations (10.5±1.3pA, and 6.0±1.1 respectively). These two sub-groups did not differ in their passive properties, their kinetics or the RMS noise in the recording (table 1). Moreover we show later that this division is kept when these cells are characterized by a different independent measure. We compared the greatly-enhanced-trained sub-group to the 4 cells with the highest averaged amplitudes in the pseudo-trained group (termed the large-pseudo sub-group) which composes the same fraction of the pseudo population. The average (figure 2B) and the standard-deviation (figure 2C) of miniature EPSCs amplitudes in this sub-group of trained neurons were 2.2 and 1.9 times larger than those in the 4 top cells in the pseudo-trained group. Given the multiplication factor of 2.3, as calculated above, this suggests that virtually all excitatory synapses in these neurons were multiplied by this factor.

**Table 1:** Neurons from the greatly-enhanced and the moderately-enhanced trained groups did not significantly differ in their membrane properties and recording conditions.

	moderately-enhanced (n = 16)	Greatly enhanced (n = 6)
**Event rise time** (**ms**)	1.32±0.29	1.46±0.50
**Event decay time** (**ms**)	4.11±1.33	5.42±1.66
**Response to −5mV step** (**pA**)	−19.35±9.68	−18.76±4.69
**RMS noise** (**pA**)	1.26±0.49	1.31±0.26

Rise-time was measured for each detected event from baseline to peak. Decay time was measured for each detected event from peak to 1/3 the amplitude of the event.

The current response was evoked at 0.16 Hz by 200 ms voltage step of −5 mV.

RMS noise was measured from 750 ms period of baseline in which no miniature events were detected.

A similar comparison between the moderately-enhanced-trained-sub-group and the pseudo-trained group, yielded a small but significant (P<0.01) increase in mean, that amounted to a factor of 1.21 only. With the multiplication factor of 2.3 this result suggests that only ∼20% of the synapses in this sub-group of neurons were multiplied by this factor after learning.

To confirm these calculations we constructed a calculated amplitude distribution curve and compared it with the experimental amplitude distribution curve: 100% of the events in the 4 cells with the highest averaged amplitude in the pseudo-trained group and random 20% of the events in the rest of the pseudo-trained cells were multiplied by 2.3. The resulting amplitude-distribution curve matched (r = 0.93) the experimental distribution curve of the trained group (figure 2D), confirming the validity of the above results. In particular, we confirmed that the averaged amplitude distribution curve of the greatly enhanced neurons can be reconstructed by multiplying all events in the 4 neurons with the highest averaged amplitudes within the pseudo cells group by a factor of 2.3. The resulting amplitude distribution curve was similar to the averaged amplitude distribution curve of the greatly enhanced group (R = 0.73; figure 2E).

These data suggest that acquisition of a skill to perform successfully in a particularly difficult task is accompanied by a comprehensive change in the strength of all synapses in a sub-population of neurons. Namely, all excitatory synapses in these cells doubled their strength.

### Synaptic enhancement is mediated by doubling the AMPA channels conductance

**Figure 3:**
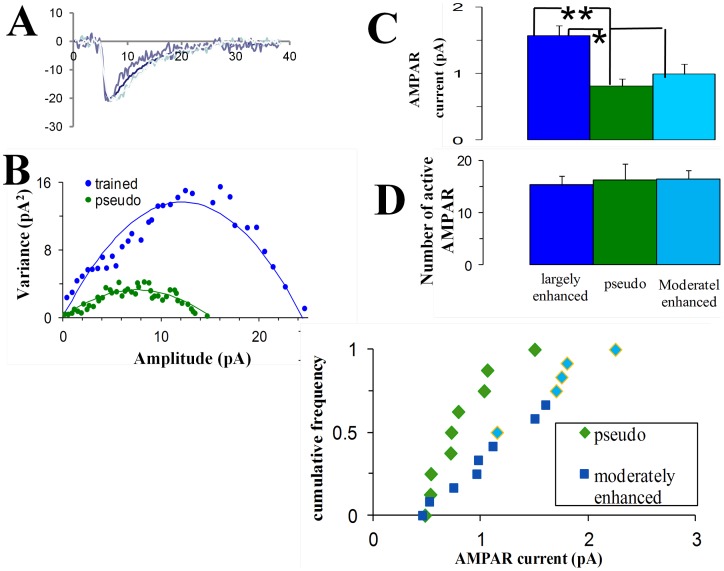
The average AMPAR-mediated conductance is doubled in the greatly-enhanced-trained-group. **A, B.** Events were peak scaled (A) and only events with rise-time <1.5 ms were used for the NSFA analysis. Variance between these peak scaled events was calculated at different time points. Current-variance plot extracted from the peak scaled mEPSC's using the NSFA analysis could be well fitted with the parabolic current-variance equation (see Methods), allowing the extraction of the averaged single channel conductance and averaged number of active AMPA channels. Examples from a pseudo-trained cell (green) and from a cell in the greatly-enhanced-trained-group (blue) are shown (B). **C.** The averaged AMPA single channel conductance in the greatly-enhanced-trained-group was doubled compared with the pseudo-trained group, and is 59% bigger than in the moderately-enhanced-trained group. Values represent mean ± SE, (**, p<0.01. *, p<0.05). **D.** The number of active AMPA channels does not differ between groups. **E.** The greatly enhanced group shows distinct values of averaged AMPAR conductance as compared with the moderately enhanced group.

Does such a twofold increase in the synaptic strength result from doubling the single channel conductance, or from doubling the number of receptors? To address this question, we applied a method that exploits the variability in event shape (non-stationary-fluctuation-analysis, NSFA, [25]–[26]), to calculate both the average single channel current and the average number of active AMPA channels in each cell (figure 3A, see Methods). The averaged calculated single channel current in the greatly-enhanced-trained-sub-group (figure 3C) was twofold higher than that of the pseudo-trained group (1.57±0.45 pA; n = 5 for trained and 0.81±0.32 pA; n = 9 for pseudo trained, P<0.0035) and was 59% higher than the averaged single channel current in the moderately-enhanced-trained-sub-group (0.99±0.43pA; n = 8, P<0.042). The calculated averaged number of active channels (figure 3D) did not differ between groups (16.3±9.0 for greatly-enhanced trained neurons, 15.3±9.0 for pseudo trained and 16.5±5.1 for moderately-enhanced trained neurons). The average single channel conductance (10 pS) calculated for the pseudo trained group is similar to that reported for hippocampal cells [23].

These results imply that multiplicative increase in mEPSC's amplitude results from a twofold increase in the AMPA-channel conductance. Furthermore, these results are in agreement with twofold increase of all synapses in the greatly-enhanced-trained-sub-group, and with a twofold increase of 20% of the synapses in the moderately-enhanced-trained-sub-group.

We divided above the neurons from the trained group to greatly-enhanced and the moderately enhanced sub-groups, based on analysis of events amplitude. The same division holds using NSFA, a method that testifies to the variability in events shape rather than events amplitudes (figure 3E), further strengthening this division.

### Whole-cell synaptic modulation enables selective memory enhancement

Calculation of the ratio of the averaged mEPSCs amplitudes between the different groups, show that synaptic events recorded only in the greatly-enhanced sub-group contribute two-third of the total learning-induced increase in the averaged mEPSC amplitude. We next examined the hypothesis that such a non-discriminatory increase in synaptic strength, observed in a quarter of the neurons in trained rats, can serve as a post-hoc mechanism to enhance Hebbian memory.

Multiplying the strength of all synapses in a cell by a constant factor has an important key feature; it does not change the relative contribution of each of the synapses. Rather, it results in multiplication of the standard-deviation of the single events amplitudes by the constant factor, and thus with multiplication of the difference between strong and weak synapses in this cell. It was shown previously that the increase in excitation is paralleled by an increase in inhibition [17]. If the averaged increase in inhibition balances the averaged increase in excitation in manner that maintains the averaged background activity unchanged, the increase in excitation alongside with an increase in inhibition will enhance the difference between strong and weak synapses without affecting the averaged activity, and thus may be termed “contrast-enhancement”. Contrast enhancement will cause the neuron to change its response to a given input such that only when the strong excitatory synapses are mostly activated, the cell will increase its firing frequency. Since activating a group of cells that form a defined Hebbian-memory will activate a large proportion of their strong excitatory synapses, contrast-enhancement will cause these neurons to increase their firing in response to this memory only, with a subtle effect on the response to partially overlapping patterns of activation representing other memories.

Thus we suggest that a whole-cell multiplication of the strength of all excitatory synapses parallel by balanced increase in inhibition can function as contrast enhancement of the cell response, and following that as a mechanism that underlies memory enhancement.

**Figure 4:**
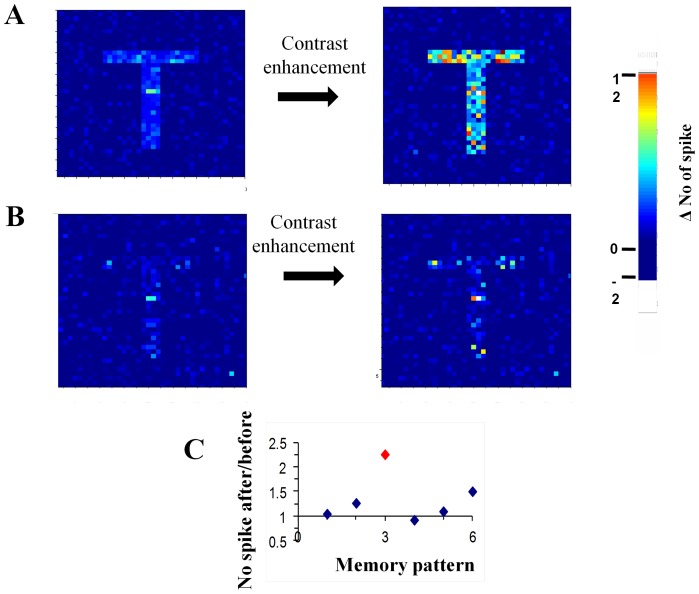
Contrast-enhancement causes selective memory enhancement. Cells in the network were arranged in a matrix in which the intensity level corresponds to the change in the number of spikes during memory activation compared to background. Cells that responded to an arbitrary input-X were arranged in T-shape. **A.** Contrast-enhancement applied to neurons constructing pattern X, substantially increased their response intensity to the input-X, but activity did not spread to neurons out of this pattern (the same neurons are activated before and after contrast enhancement is applied). **B.** Contrast enhancement of pattern X did not affect the intensity of another memory-pattern induced by a different input, although the two memory-patterns had a considerable overlap (correlation of 0.74). Notice the vague shape of the T pattern. **C.** The ratio between the number of spikes before and after contrast-enhancement in response to different inputs. Only the response to input-X (red) was considerably enhanced. Pattern #6 is shown in figure 4B.

This potential function of contrast-enhancement can be studied with a biophysical neuronal network simulation. To that aim, we constructed a neuronal network that entails 2800 excitatory and inhibitory neurons, in which neurons exhibit background activity, and memory is formed by additional activation of external inputs on 28% of the cells in the network (see Methods). As first step, the network “learned” 6 patterns by Hebbian-learning. Subsequently, we selected a group of cells that exhibited a significant (P<0.02) increased firing frequency in response to activation of one of the input-patterns (designated as X-pattern). On all cells in this group we applied contrast enhancement such that we multiplied the strength of all excitatory synapses by a factor of 2.5 and multiplied the strength of all inhibitory synapses by a constant factor such that the background activity will maintain the same value. We compared the response of these cells to the activation of each pattern before and after contrast-enhancement. The response induced by activation of the X-pattern was markedly enhanced by the contrast-enhancement (figure 4A, C), while the response to activation of other patterns was not significantly affected (figure 4B, C).

In our model we increased the inhibition by a multiplication process in which the strength of all synapses in all selected cells was multiplied by the same constant. Such a multiplication process is simple since unlike an additive process it does not require knowledge of the total strength of the excitatory synapses and of the inhibitory synapses. We found that in order to maintain the resting activity of the cells after contrast enhancement, the multiplication constant averaged over the whole sets of simulation was amounted to 2.2±0.3. Indeed the same multiplication factor for the inhibition and excitation will not modify the synaptic reversal potential, and thus will allow the same resting activity.

A memory enhancement that is based on a contrast enhancement mechanism in which the strength of all excitatory synapses and inhibitory synapses is multiplied by a constant does not have a synaptic specific memory and thus it can be applied as a whole cell process that can be switched on and off when necessary.

### Model predictions

Our whole-cell memory enhancement model leads to several testable predictions;

**1.** Contrast-enhancement requires that both excitation and inhibition should be simultaneously enhanced. Moreover inhibition should be enhanced predominantly in the greatly enhanced group in order to balance the predominant increased excitation in this group. We previously showed that following learning the increase in excitation is paralleled by an increase in inhibition and that the inhibition is predominantly increased in a sub-group of cells [17].

**2.** Contrast-enhancement requires that the increase in excitation will be balanced by an increased inhibition such that the cell response during background activity will not be modified by contrast-enhancement. Keeping the cell in a balanced state would enable the cell to continue generating memory-relevant patterns of activation. Thus the model predicts that the increase in inhibition will be to the same degree as the increase in excitation.

**3.** A mechanism that does not require a synaptic specific memory does not require synaptic tagging and thus can be readily switched on and off when necessary. We showed that the increased excitation does not require a synapse specific memory since it is a whole cell process that acts uniformly on the cell's excitatory synapse population by multiplication of the strength of the synapse by the same constant. We predict that in order to remain synapse unspecific and in order to maintain the balance between excitation and inhibition the increase in inhibition should be also mediated by a whole-cell process in which the GABA_A_ synaptic strength is doubled.

**4.** Multiplication of the synaptic strength in each synapse can be mediated through an increase of the number of channels or through an increase of the channel conductance.

Increasing the inhibition through multiplication of the number of channels would require knowledge of the number of channels in each synapse. A process that acts directly on the synaptic channel conductance is simpler, since it does not require knowledge of the number of channels. We indeed had shown that contrast enhancement acts through doubling the AMPA channel conductance and predict that the increased inhibition will also be mediated by a multiplicative process in which the GABA_A_ channel conductance is doubled.

To test these predictions we had analyzed TTX-insensitive pure GABAA-mediated miniature-inhibitory-post-synaptic-currents (mIPSCs) that were recorded from layer II pyramidal neurons 4–5 days after learning-set.

### Synaptic enhancement is dominated by doubling of GABA_A_-mediated currents

We recently showed [17] that the increase in excitation was paralleled by a robust increase in the amplitude of miniature IPSC's (35%), and that the increase in inhibitory events amplitude was predominant in a sub-group of cells, were virtually all inhibitory events increased their strength (Averaged mIPSC amplitude 23.8±7.0 pA in neurons from trained rats, 20.9±2.9 pA in neurons from naïve rats, and 18.2±3.9 pA in neurons from pseudo-trained rats). Here we examined the other prediction of the models, namely that in these cells the increase is controlled by a uniform all-cell process in which the conductance of the GABA_A_ channel is doubled.

**Figure 5:**
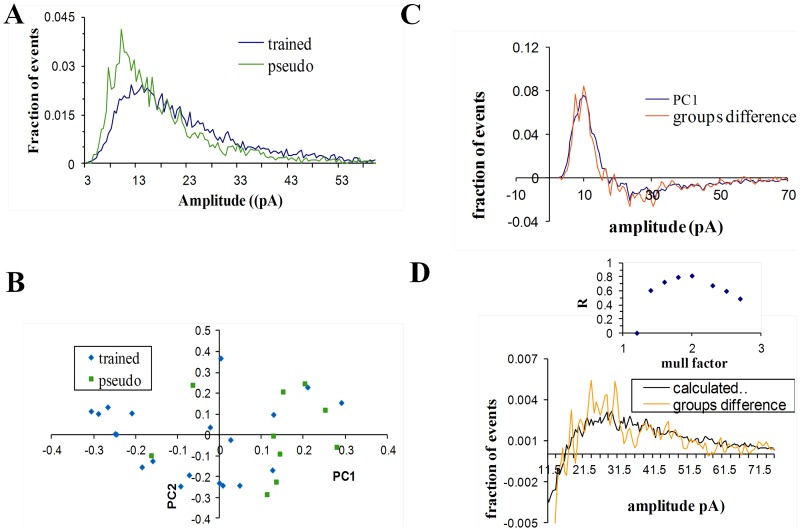
The learning-induced modulation of the inhibitory unitary synaptic events amplitude is dominated by a single process in which the events amplitude was doubled. **A.** For each neuron an amplitude distribution curve was reconstructed from the mIPSC's amplitudes and was normalized (data taken form [17]). Averaged amplitude histograms, were calculated for all neurons from the three groups. Notably, a significant portion of events in the trained group are of higher values. **B.** For each cell the weight for the first component (X-axis) was drawn against its weight for the second component (Y-axis). Only the weights of the first component are significantly different between groups. **C.** The curve calculated by subtracting the averaged pseudo amplitude distribution curve from the averaged trained amplitude distribution curve (orange) match the first component calculated by PCA (black). **D.** For each multiplication factor a different curve that describes the difference between groups assuming a multiplication model was calculated. The calculated curve assuming multiplication factor of 2 (black) matched the curve that describes the main difference between groups (orange). Inset: the correlation coefficient was calculated for each multiplication factor (for calculation of R only amplitudes >13pA were used, since at lower amplitudes, multiplication factors bigger than two requires unavailable data in amplitudes <6pA, see Methods).

We characterized the process that underlies the increased inhibition by analyzing the amplitude distribution curves of the previously measured [17] inhibitory events. The averaged amplitude distribution curves of neurons from trained rats picked at higher amplitudes and decayed at lower rates, compared with the averaged values for neurons in the pseudo trained group (figure 5A).

We next examined the process that underlies the increased averaged amplitude of inhibitory events. We Applied PCA on all distribution curves from pseudo and trained groups. The first component could explain 38% of the difference between all distribution curves; the second component could explain 10% of the differences while the third and higher components could explain less than 4%. Post-hoc examination of each group revealed that the weights of the first component in cells from the trained group (−0.06±0.17, n = 20) was significantly different (P<0.008; figure 5B), compared to the pseudo-trained group (0.12±0.13 n = 10) while the weights of the other components did not differ significantly between groups (P<0.7). Together this suggests that the first component describes the main difference between the groups while the other components describes within group differences. Moreover, the first component well matched the curve describing the averaged difference between groups (R = 0.93, figure 5C) suggesting that one process dominates the difference between the two groups.

Subsequently, we tested whether the process underlies the difference between group is multiplicative or additive, as shown for excitatory synaptic transmission. With multiplication factors between 1.8–2, the curve that describes the main difference between the groups well matched the calculated curve assuming a multiplicative model (figure 5D, see also Methods). An alternative model, assuming a process of addition rather than multiplication (in which all synapses are increased by a constant), resulted in poor fit (R = 0) for all constants.

### In a sub-group of neurons the strength of all inhibitory synapses is doubled

**Figure 6:**
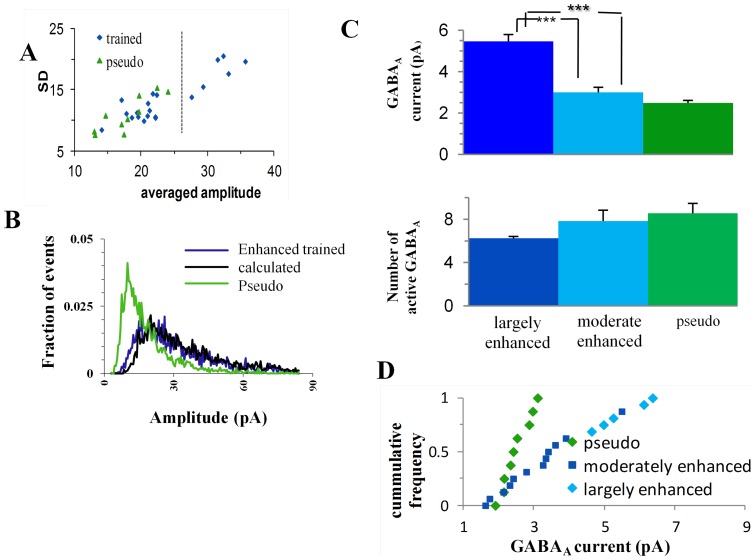
In a sub-group of cells from trained rats, amplitudes of all inhibitory miniature events are doubled. **A.** Each cell was plotted as a function of its averaged event amplitude and standard deviation. Few cells from trained-group had exceptionally large averaged amplitudes and Standard-deviations. Using hierarchical clustering analysis the cells were divided to two groups (separated by the dotted line; the same division was obtained for all the methods that were implemented). **B.** The distribution curve describing the greatly-enhanced trained neurons can be constructed from the distribution curve describing the pseudo-trained neurons. The expected curve (black) calculated from pseudo events (green) overlaps the averaged distribution curve of the greatly-enhanced-trained group (blue). (r = 0.85, only amplitudes >13pA were used, since at lower amplitudes, multiplication factors bigger than two requires unavailable data in amplitudes <6pA, see Methods) **C.** The averaged GABA_A_ single channel conductance in the greatly-enhanced-trained-group was doubled compared with the pseudo-trained group, and is 82% bigger than in the moderately-enhanced-trained group. Values represent mean ± SE, (***, p<0.001). The number of active GABA_A_ channels does not differ between groups. **D.** The greatly enhanced group shows distinct values of averaged GABA_A_R conductance as compared with the moderately enhanced group.

The increase in mIPSC amplitude and in SD was not homogenous and some neurons showed a particular strong increase both in amplitude and SD [17]. The increase in standard deviation in the trained group was similar to the increase in amplitude (figure 6A). Using hierarchical analysis [24], the neurons in the trained group could be segregated into two distinct sub-groups (figure 6A). The first sub-group, the greatly-enhanced neurons, entails six cells (30% of the trained group) with very large means (31.6±2.8 pA) and large standard-deviations (17.9±2.0). The second sub-population, the moderately-enhanced neurons (all other 15 cells) had significantly (P<0.004) lower means and standard-deviations (19.8±2.3pA, and 11.4±1.6 respectively). The cells in the greatly enhanced group are those in which most of the synapses showed an increase [17]. We compared the greatly-enhanced-trained sub-group to the cells in the pseudo-trained group. The average and the standard-deviation in this sub-group of trained neurons were 1.79 and 1.69 times larger than those in the pseudo-trained group. Given the multiplication factor of 2, calculated using the PCA component, this suggests that virtually all inhibitory synapses in these neurons were multiplied by this factor.

The averaged amplitude of events in the moderately enhanced sub-group was not significantly different than the averaged amplitude in neurons from the pseudo group (19.8±2.3pA, and 18.2±3.9 pA respectively increase of 1.1; P<0.2). Calculation of the ratio of the averaged mIPSCs amplitudes between the different groups, show that synaptic events recorded only in the greatly-enhanced sub-group contribute more than two-third of the total learning-induced increase in the averaged mIPSC amplitude.

We tested the hypothesis that in the greatly enhanced group all inhibitory events were doubled by comparing the calculated curve with the experimental curve: all events in the pseudo-trained group were multiplied by 2, and an amplitude distribution curve was calculated. The resulting amplitude-distribution curve matched (r = 0.85) the experimental distribution curve of the greatly enhanced trained group (figure 6B), confirming the validity of the above results.

These data suggest that acquisition of a skill to perform successfully in a particularly difficult task is accompanied by a comprehensive change in the strength of all inhibitory synapses in a sub-population of neurons. Namely, all inhibitory synapses in these cells doubled their strength.

### Synaptic enhancement is mediated by doubling the GABA_A_ channels conductance

To determine whether the twofold increase in the synaptic strength result from doubling the single channel conductance or from doubling the number of receptors, we applied the NSFA analysis method. As shown for excitatory synaptic transmission, the averaged calculated single GABA_A_ channel current in the greatly-enhanced-trained-sub-group (figure 6C) was twofold higher than that of the pseudo-trained group (5.46±0.74 pA; n = 5 for greatly enhanced trained and 2.48±0.41 pA; n = 9 for pseudo trained, P<0.00001) and was 82% higher than the averaged single channel current in the moderately-enhanced-trained-sub-group (2.99±1.07pA; n = 11, P<0.0003). The calculated averaged number of active channels (figure 6C) did not differ between groups (6.3±0.4 for greatly-enhanced trained neurons, 8.6±2.8 for pseudo trained and 7.8±2.5 for moderately-enhanced trained neurons). These results imply that multiplicative increase in mIPSC's amplitude results from a twofold increase in the GABA_A_-channel conductance. Furthermore, these results are in agreement with twofold increase of all inhibitory synapses in the greatly-enhanced-trained-sub-group. As for excitatory synapses, the division between greatly enhanced and moderately enhanced groups was kept when taking the single channel conductance as a parameter (figure 6D).

## Discussion

We have previously shown that complex odor-learning is accompanied by pronounced, wide spread, enhancement of excitatory and inhibitory synaptic transmission within the piriform cortex mediated by post synaptic modulation of AMPA receptor and GABAA receptor mediated currents [17]. Here using in-depth analysis, we show that the increase of the post synaptic currents is due to increase in the receptors channels conductance and suggest that most of the increase is attributed to doubling the strength of all synapses in a sub-group of cells. This large scale synaptic modulation may be instrumental for enhancing long-term Hebbian-memory of highly-complex skills. Such memory enhancement does not require synapse-specific memory and is independent of the number of channels and thus can be readily switched on and off when necessary.

Using network simulations, we show how such whole-cell modification in excitatory synaptic transmission sub serves as a transient mechanism for selective memory enhancement. Using the model we show that learning-induced modifications in inhibitory synaptic transmission must exist alongside with changes in synaptic excitation. Moreover, the mechanism underlying modulation of inhibitory spontaneous synaptic events is predicted accurately by model.

### Excitatory synaptic enhancement is mediated by post synaptic modulation of the AMPAR conductance

We show, using the PCA analysis, that most of the differences between the pseudo and trained groups are caused by doubling the event size. We validated this using the NSFA and further showed that the two-fold increase of the AMPA channel conductance, and not a change in the averaged number of active channels per unitary synaptic event, underlies the two-fold increase in the event size. We claim that a modification in the channel conductance and not in the number of channels can serve as a transient mechanism that does not require knowledge of the synaptic strength and thus can be served as a mechanism that can be readily switched on and off. Interestingly, it was found that phosphorylation of ser-831 site of the AMPA receptor causes doubling of the AMPA channel conductance [27] and that this increase follows synaptic stimulation and returns to baseline values after depotentiation [21], [22]. The increase we observed in the averaged events amplitude could not be explained by increase in the AMPA channel open time since it did not resulted in differences in the events kinetics (table 1).

The conductance of an AMPA channel reflects the number of sub-units that are simultaneously conducting. After phosphrilation of ser-831 the relative proportion of simultaneously open sub-units is increased and thereby increasing the averaged conductance of the AMPA channel. The relative proportion of the sub-conductance's is stable both before and after phosphorilation, leading to two stable conductance states with the ratio of two [28]. As a result, the scaling of the amplitude of a single excitatory event after ser-831 phosphorilation should be maximum two. This together with our observations that the events amplitudes and the average channel conductance were also multiplied by two indicates that a single multiplication factor of around two is indeed expected for all events.

### Greatly-enhanced versus moderately-enhanced neuronal groups

Although learning-induced synaptic enhancement is apparent in most recorded neurons, extensive changes are present in a sub-group of neurons, termed the greatly-enhanced cells, in which effectively all synapses doubled their strength.

Although the greatly-enhanced trained sub-group entails only a relatively small fraction of the pyramidal cell population, it contributes two-thirds of the total increase in the averaged mEPSC amplitude.

The dominance of the greatly-enhanced cells, in which almost all synapses double their strength, implies that the synaptic connectivity in all the pathways to and within the piriform cortex should all increase by a similar factor. Indeed, enhanced excitatory transmission was observed after learning in the ascending and descending fibers terminating on layer II pyramidal neurons [15], as well as in the intrinsic fibers inter-connecting these neurons [16], [17], [29]; This increase had a similar magnitude (∼60%) in all pathways. Moreover, the extent of increase observed in these pathways (∼60%) is similar to the extent of increase in synaptic strength observed here. This consistency is mainly attributed to the dominant contribution of the greatly-enhanced-trained-sub-group to the synaptic responses evoked in all pathways.

### Possible implications of whole-cell AMPAR conductance multiplication

Since the enhancement we observed is cell-specific, rather than synapse-specific, the conductance increase can be mediated by whole-cell control mechanism(s). Several studies report a robust long-term increase in the total synaptic strength following learning [9]–[13], [15]. The increase in excitation and in inhibition was observed 4–5 days after training termination [17]. We previously showed that the long term increase observed in our lab is paralleled by only minor morphological modifications [18]. A whole-cell transduction mechanism can indeed support the observed long-term change without morphological support. Moreover, the increase in synaptic strength was shown to be transient, disappearing within eight days after training termination [9], [12]. Indeed, a whole-cell process that is meditated by transduction mechanism and is not supported by morphological modifications can be toggled off, and thus to cause the synaptic strength to resume its baseline values.

### Long-lasting enhancement of inhibitory synaptic transmission

The validity of our computational model could be readily examined by testing the four predictions it generates regarding the learning-induced long-term modulation of synaptic inhibition: (1) the increase in inhibition should be dominant in a sub-group of cells. Indeed the predominant increased inhibition in the greatly-enhanced group could explain two-third of the total increase in inhibition. (2) The increased synaptic inhibition should be of the same extent as synaptic excitation. Indeed, amplitude of both miniature inhibitory and excitatory events was increased by approximately two-fold both for inhibitory and excitatory events. (3) The increased inhibition should be mediated by a whole-cell process in which the increase is uniform over the synapse population. Our data show, using different analysis methods, that virtually the amplitudes of all inhibitory events in the greatly enhanced group are doubled. (4) The mechanism that mediates multiplication of the inhibitory synaptic strength should act on the GABA_A_ channel conductance rather than the number of channels. Indeed, using NSFA analysis we showed that the average number of GABA_A_ channels per event was not modified while the channel conductance was modified.

### Contrast-enhancement

Pyramidal cells in the piriform cortex were found to be electrotonically compact with space constant of current transmission that approximates 900 µm [30], thus enabling the quantification of both proximal and distal generation sites.

We suggest that a multiplicative increase of all excitatory and inhibitory synapses in the cell is a novel whole-cell mechanism for selective enhancement of Hebbian memory, which is achieved through a process we termed “contrast-enhancement”.

Multiplication of the inhibition and excitation by the same constant will amplify the net synaptic current without modifying its reversal potential. This should cause a prominent increase in spike rate mainly when the net synaptic is considerably depolarized, thus mainly when the cell is part of a memory pattern. When the cell does not respond to the input, the net synaptic current is small and thus the absolute change caused by contrast enhancement should be minor. When the input has an inhibitory effect on the cell and the net synaptic current is hyperpolarizing, contrast enhancement will further hyperpolarize the cell voltage. This implies that a multiplicative increase by the same constant of both inhibition and excitation only scales the cell response without modifying its quality and thus can be termed contrast enhancement.

Notably, contrast enhancement could not be mediated by an additive process, in which all excitatory synapses are increased rather than multiplied by a constant factor. Such an additive process is expected to have a minor effect on the difference between a responding state and non responding state.

### Functional significance of contrast-enhancement

Using a neuronal network model, we showed that applying contrast-enhancement on a group of cells that forms a distinct memory leads to selective enhancement of this particular memory, with a minor effect on other memories that are stored in the same network. Such memory enhancement is independent of memory formation. A memory of vital importance needs to be enhanced in order to dominate subsequent behavior [14], [31]. Examples for such a memory can be found in reward related memory [14], [32] or in traumatic memory [31]. When the memory becomes less crucial it should be de-enhanced in order to balance its weight with the weights of other memories. The balance between memories in a network of neurons is achieved through a tight upper limit of the synaptic strength [33–35). The limit is hypothesized to occur via a limit on the number of AMPA receptors [36]. Contrast-enhancement bypasses the tight control of synaptic strength by increasing the AMPA channel conductance rather by increasing the number of AMPA receptors; it is cell-specific rather than synapse-specific and thus has the potential to be readily toggle-on and off via whole-cell transduction mechanisms.

To conclude, our study shows that high-skill learning induces a profound long-lasting modulation of AMPAR and GABA_A_R conductance-mediated enhancement of excitatory and inhibitory synaptic transmission. This enhancement is mostly induced in a sub-group of neurons, in which virtually all synaptic inputs double their strength. Such a whole-cell modulation enables the cortical network to enhance particularly important memories, on the background of other, somewhat overlapping, memories. We suggest that this unique memory enhancement mechanism in crucial for maintaining recently acquired capabilities to perform particularly complex tasks.

## Materials and Methods

### Statistical analysis

Between-groups comparison was done using one-way ANOVA, and post-hoc multiple t-tests were then applied to compare between each two groups. Values throughout the text are presented as mean ± SD. Data in graphs is presented as mean ± SE.

### PCA

We applied principal component analysis (PCA) on the amplitude distribution curves of all cells in the trained and the pseudo-trained group, after subtracting the total average.

PCA is a model-free analysis method that approximates a data set with a linear combination of a small number of untailored components. The method gives an importance score to each component, such that the first component has the highest score.

The weights of the linear combination are different for each cell. The method has no knowledge which cell was pseudo-trained and which was trained in this pool.

### Calculating the model based curve that describes the difference between groups

Given a multiplication factor, the fraction of events that has to be multiplied by this factor in order to result with the averaged increase in event amplitude (64% for excitation and 35% for inhibition) was calculated. A new amplitude distribution curve was calculated by randomly selecting the calculated fraction of the events in the pseudo group and multiplying their amplitude by this factor. The averaged pseudo amplitude distribution curve was then subtracted from the calculated amplitude distribution curve.

Only amplitudes >7pA for excitation and >13pA for inhibition were used for the calculation of the correlation coefficient since the construction of the model-based element at lower amplitudes needs unavailable data at amplitudes <3 pA for excitation and <6 for inhibition, when multiplication factors >2.5 are used.

### NSFA

Estimate of the averaged single channel current and the averaged number of active channels were obtained using a peak-scaled non-stationary fluctuation analysis (NSFA) of mEPSC'c [21]. The NSFA was applied on the events that were electrotonically nearby (10–90% rise-times <1.5 ms; 61±14% of events in trained; 64±16% in pseudo-trained). Using Mini analysis software (Synaptosoft Inc.), events (80–300 per each cell) were scaled and aligned by their peak, and their decay phase was divided to 30 bins. The single channel current (i) and the number of channels (N) were calculated (using Mini Analysis Software) by fitting the theoretical relationship for the peak scaled variance (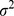) after subtraction of the background variance (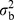):
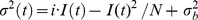



Only cells in which the fitting of the equation yielded an R>0.85 were incorporated in the analysis.

The Single channel conductance (γ) was calculated using the equation
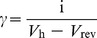
where V_h_ is the holding potential (−80 mV) and V_rev_ is the reversal potential of the AMPA channel ( 0 mV).

### Network Modeling

The network is composed from N_E_ excitatory and N_I_ inhibitory integrate-and-fire current-base neurons. The sub-threshold activity of each neuron is described by the following equation:
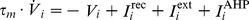
(1)where the subscript i refers to the neuron number; 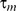 is the membrane time constant; 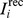 is the sum of the recurrent synaptic input from neurons in the network; 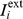
is the sum of synaptic currents from external neurons in other brain areas; 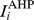
is the After Hyper Polarization (AHP) current activated after every spike. Membrane resistance has been absorbed into the definition of the currents in Eq. 1.

Whenever the depolarization of a neuron hits a fixed threshold θ (V_i_(t) >θ), the neuron emits a spike, causing all its output synapses to be activated, yielding a synaptic current on the postsynaptic neurons within a uniformly distributed period of 0.3–4 ms (D_ij_). After a spike, V_i_ is reset to zero and then resumes its pre-spike integration, followed by AHP current.

Excitatory neurons were randomly interconnected. Inhibitory and excitatory neurons were also randomly inter-connected.

The external input 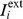
is governed by homogenous Poisson process and is composed of continuously activated synapses 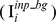
supplying random background input which led to a background activity with average of 1 spikes/sec and from inputs that were activated selectively 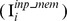
and drove the network response. 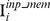
was applied on 28% of the excitatory cells. Its activation rate varied during the course of the trial such that it was silent during the first and the last 1000 msec of the trial. We implemented feed-forward inhibition such that the external input activated also the inhibitory cells.

The recurrent current 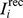
is the sum of the postsynaptic currents from all neurons in the network targeting neuron i:
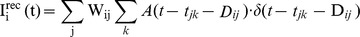
Where 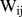
is the efficacy of the synapse connecting neuron j to neuron i; A(t) is the time course of the postsynaptic current, governed by instantaneous rise and a single exponential decay; the sum on k is over all the emission times 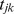
of presynaptic neuron j; 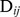 is the transmission delay.

A memory was learned by changing the strength of the relevant synapses. The strength of the connectivity between neurons, W_ij_, was modulated based on Spike-Timing-Dependent-Plasticity (STDP) rule [37]. The basic rule for STDP-based changes is given by:


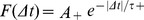
 if Δt >0; 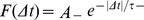 if Δt <0Where Δt is the timing of the post-synaptic spike relative to the pre-synaptic spike.

We applied boundary conditions on the synaptic strength, such that the size of the synaptic change F(Δt) is reduced as W_ij_(t) approaches its limits [35] and achieve it by multiplying F(Δt) by the equation :


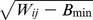
 if Δt <0; 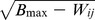 if Δt >0where B_min_ and B_max_ are the lower bound and the upper bound respectively.

A great deal of care was taken to keep the activity of the neurons in a Poisson fashion. We achieved this by choosing the right initial values for the network, and later by controlling the rate of learning. In addition we implemented activity-dependent scaling of synaptic strength, modeling the phenomena reported in pyramidal neurons cultured from rat visual cortex [38]. Post-synaptic activity in each excitatory neuron j was tracked by counting the number of spikes (a_j_) during the last 1000 msec of a trial in which I_i_^inp_mem^ was silent. If the activity of the neuron wandered outside the range of (a_min,_ a_max_), synaptic scaling was applied at the end of the trial by adding the product of the following equation to W_ij_:


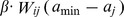
 if 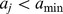; 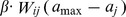 if 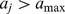;

The synaptic scaling was applied on all excitatory and inhibitory synapses in the cell excluding the synapses of the background activity.

Each cell had 25 afferent input synapses. In each memory activation 20% of the synapses were activated on each cell.

Memories were learned by repeating the same input during 20 trials. In each trial, input was applied for 1 sec. For each memory, randomly chosen 28% of cells received afferent input. Values for all the parameters that were used in the simulations are listed at Table 2.

**Table 2:** Values for simulation parameters.

Single-cell parameters	
τ_m_ – membrane time constant	9 ms
θ – spike threshold	1 mV
τ_e_ – decay time constant of excitatory current	4 ms
τ_i_ – decay time constant of inhibitory current	6 ms
**Network parameters**	
N_E_ – number of excitatory cells	2000
N_I_ – number of inhibitory cells	800
Probability of E → E synaptic contact	0.2
Probability of E → I synaptic contact	0.05
Probability of I → E synaptic contact	0.015
Initial synaptic efficacy E → E	0.0075 mV
synaptic efficacy E → I	0.04 mV
synaptic efficacy I → E	−0.06 mV
%cells receiving excitatory input	28%
%cells receiving inhibitory input	56%
Initial excitatory input	0.015 mV
Inhibitory input	0.006 mV
Input frequency	10 hZ
**Learning parameters**	
A_+_ learning rate	0.003
A_−_ learning rate	−0.0015
τ^+^ time constant of STDP window	13 ms
τ^−^ time constant of STDP window	26 ms
B_min_ – lower bound for synaptic size	0 mV
B_max – upper bound_	0.045 mV
β – rate of synaptic scaling	0.15
a_min ,_ a_max_ – lower and upper bound on spike count.	0.15 Hz; 3.5 Hz

The parametrs listed in the tables underly the basic single cell properties, network connectivity and learning.

### Applying contrast enhancement

The foot print of a memory in a network context is the response of the cells to I_i_^inp_mem^. For each neuron i, contrast enhancement was applied if the response to I_i_^inp_mem^ was significantly (P<0.02) different from its response to background activity over 10 trials. In each cell in which contrast-enhancement was applied, all excitatory synapses (both recurrent and afferent) were multiplied by 2.5, the same fraction of inhibitory synapses were multiplied by a factor that was derived such that the averaged activity of all neurons that were modulated by the contrast enhancement will remain the same (in our simulation the value was of 2.2±0.3). Typically contrast enhancement was applied on ∼5% of the cells for single memory activation.

Simulations were conducted using the event driven simulation implemented in Neuron [39].

## Supporting Information

Figure S1 The second PCA component describes the inner variability within groups, whereas the inner variability in the trained group is two-fold expansion in the X-axis of the inner variability pseudo group. The weights of PC1 and PC2 are well correlate both for the pseudo and trained groups and therefore the same linear combination of the two principal components is sufficient to describe all distribution curves in the same group. **A.** The first two components (PC1 and PC2) calculated by Principal Component Analysis on the pool of mepsc's distribution curves from pseudo and trained groups. **B.** The compound component of the pseudo group (red) was build based on the correlation between the weights of PC1 and PC2 for the pseudo group (figure 1D; PC1+0.88·PC2). The curve describing the main variability within the pseudo group (blue) was obtained by applying PCA on the pseudo group only. The resulting first PC well overlapped the compound component (r = 0.77). **C.** The compound component of the trained group (red) was build (PC1-0.8·PC2). PCA was applied on the trained group only, where the resulting first PC (blue) describes the main variability within the trained group. The first PC well overlapped the compound component (r = 0.80). **D.** The curve calculated by multiplying the compound pseudo component in the X-axis by a factor of 2.2 describes well (r = 0.86) the compound component of the trained group (for calculation of R only amplitudes >13pA were used, since at lower amplitudes, multiplication factors bigger than two requires unavailable data in amplitudes <6pA, see Methods).
(TIF)

Table S1 When the difference between groups is moderate, a good correlation between the curve describing the averaged differences between groups and PC1 is attained only if big majority of the events were multiplied by the same factor, or if the multiplication factors had similar values. The distribution curves of the pseudo group were modified with different multiplicative transformations and then normalized. PCA analysis was calculated on a pool of distribution curves containing the pseudo and the transformed data. For each transformation, the correlation coefficient (**r**) between PC1 and the curve that resulted from subtracting the pseudo mean curve from the transformed mean curve was calculated. In addition the significance value between the weights of the two groups was calculated both for PC1 and PC2 (**PC1, PC2**). Only 50% of the mepsc's population was modified. The modified group was divided such that each portion (X, Y) was multiplied by a different multiplication factor(X*a, Y*b). The proportions were chosen such that the weighted average of the multiplication factors will be 2.5.
(DOCX)

Table S2 When the difference between groups is large (>150%), a high correlation is attained even though the multiplication factors are significantly different. The distribution curves of the pseudo group were modified with different multiplicative transformations and then normalized. PCA analysis was calculated on a pool of distribution curves containing the pseudo and the transformed data. For each transformation, the correlation coefficient (**r**) between PC1 and the curve that resulted from subtracting the pseudo mean curve from the transformed mean curve was calculated. In addition the significance value between the weights of the two groups was calculated both for PC1 and PC2 (**PC1, PC2**). Half of the population was multiplied by **a** and half by **b**, causing more than two-fold difference between groups. A high correlation is attained even though the multiplication factors were very different.
(DOCX)

Table S3 When both additive and multiplicative processes underlie the difference between groups, a good correlation is attained only if one of the processes is very minor. The distribution curves of the pseudo group were modified with different transformations and then normalized. PCA analysis was calculated on a pool of distribution curves containing the pseudo and the transformed data. For each transformation, the correlation coefficient (**r**) between PC1 and the curve that resulted from subtracting the pseudo mean curve from the transformed mean curve was calculated. In addition the significance value between the weights of the two groups was calculated both for PC1 and PC2 (**PC1, PC2**). Two different processes were applied, an additive and a multiplicative process. The amplitude of 25% of the mepsc's population was increased by factor **a** and the amplitude of other 25% of the population was multiplied by factor **b**.
(DOCX)
