# Supplementary figures and images for: A Novel Whole-Cell Mechanism for Long-Term Memory Enhancement
Source: PLoS One. 2013 Jul 11;8(7):e68131. doi: 10.1371/journal.pone.0068131 (PMC3708920; doi:10.1371/journal.pone.0068131)

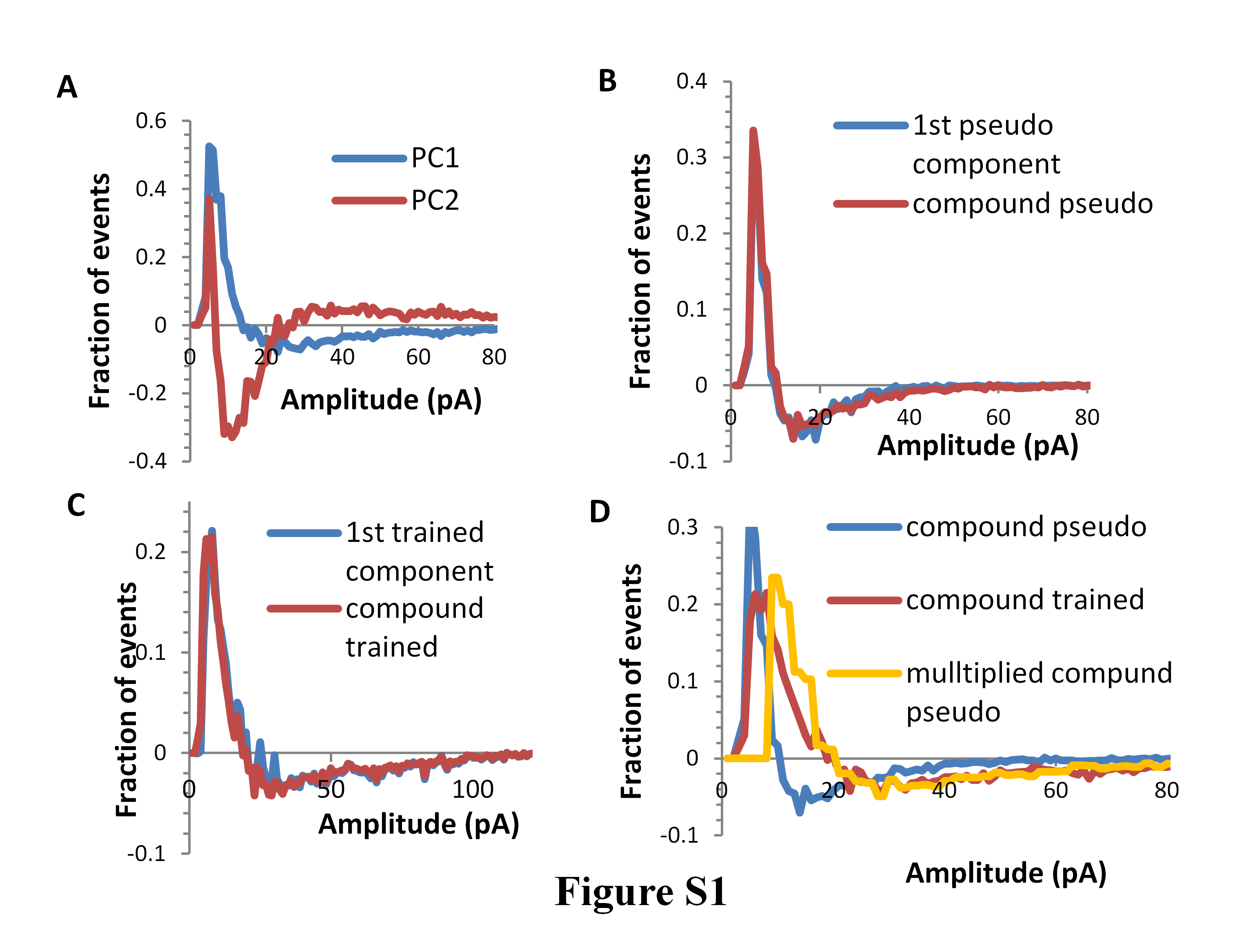

Supplement: Figure S1 — The second PCA component describes the inner variability within groups, whereas the inner variability in the trained group is two-fold expansion in the X-axis of the inner variability pseudo group. The weights of PC1 and PC2 are well correlate both for the pseudo and trained groups and therefore the same linear combination of the two principal components is sufficient to describe all distribution curves in the same group. A. The first two components (PC1 and PC2) calculated by Principal Component Analysis on the pool of mepsc's distribution curves from pseudo and trained groups. B. The compound component of the pseudo group (red) was build based on the correlation between the weights of PC1 and PC2 for the pseudo group (figure 1D; PC1+0.88·PC2). The curve describing the main variability within the pseudo group (blue) was obtained by applying PCA on the pseudo group only. The resulting first PC well overlapped the compound component (r = 0.77). C. The compound component of the trained group (red) was build (PC1-0.8·PC2). PCA was applied on the trained group only, where the resulting first PC (blue) describes the main variability within the trained group. The first PC well overlapped the compound component (r = 0.80). D. The curve calculated by multiplying the compound pseudo component in the X-axis by a factor of 2.2 describes well (r = 0.86) the compound component of the trained group (for calculation of R only amplitudes >13pA were used, since at lower amplitudes, multiplication factors bigger than two requires unavailable data in amplitudes <6pA, see Methods). (TIF) [file pone.0068131.s001.tif]
