# Supplementary material for: A Novel Whole-Cell Mechanism for Long-Term Memory Enhancement
Source: PLoS One. 2013 Jul 11;8(7):e68131. doi: 10.1371/journal.pone.0068131 (PMC3708920; doi:10.1371/journal.pone.0068131)
Supplement: Table S1 — When the difference between groups is moderate, a good correlation between the curve describing the averaged differences between groups and PC1 is attained only if big majority of the events were multiplied by the same factor, or if the multiplication factors had similar values. The distribution curves of the pseudo group were modified with different multiplicative transformations and then normalized. PCA analysis was calculated on a pool of distribution curves containing the pseudo and the transformed data. For each transformation, the correlation coefficient (r) between PC1 and the curve that resulted from subtracting the pseudo mean curve from the transformed mean curve was calculated. In addition the significance value between the weights of the two groups was calculated both for PC1 and PC2 (PC1, PC2). Only 50% of the mepsc's population was modified. The modified group was divided such that each portion (X, Y) was multiplied by a different multiplication factor(X*a, Y*b). The proportions were chosen such that the weighted average of the multiplication factors will be 2.5. (DOCX) [file pone.0068131.s002.docx]

| **Multiplication factors(a, b)** | **2.5; 2.5** | **3, 2** | **4, 2** | **5, 2** | **6, 2** | **8, 2** | **10, 2** | **20, 2** | **1.5, 5** | **1.5, 4** | **1.5, 3.5** |
| --- | --- | --- | --- | --- | --- | --- | --- | --- | --- | --- | --- |
| **r** | 0.77 | 0.70 | 0.62 | 0.61 | 0.53 | 0.55 | 0.62 | 0.77 | 0.5 | 0.59 | 0.61 |
| **PC1** | ** | * | * | * | * | * | ** | ** | ** | ** | ** |
| **PC2** | - | - | * | * | * | ** | * | ** | * | - | * |

**Table S1: When the difference between groups is moderate, a good correlation between the curve describing the averaged differences between groups and PC1 is attained only if big majority of the events were multiplied by the same factor, or if the multiplication factors had similar values.**

The distribution curves of the pseudo group were modified with different multiplicative transformations and then normalized. PCA analysis was calculated on a pool of distribution curves containing the pseudo and the transformed data. For each transformation, the correlation coefficient (**r**) between PC1 and the curve that resulted from subtracting the pseudo mean curve from the transformed mean curve was calculated. In addition the significance value between the weights of the two groups was calculated both for PC1 and PC2 (**PC1, PC2**).

Only 50% of the mepsc’s population was modified. The modified group was divided such that each portion (X, Y) was multiplied by a different multiplication factor(X*a, Y*b). The proportions were chosen such that the weighted average of the multiplication factors will be 2.5.
