# Supplementary material for: A Novel Whole-Cell Mechanism for Long-Term Memory Enhancement
Source: PLoS One. 2013 Jul 11;8(7):e68131. doi: 10.1371/journal.pone.0068131 (PMC3708920; doi:10.1371/journal.pone.0068131)
Supplement: Table S2 — When the difference between groups is large (>150%), a high correlation is attained even though the multiplication factors are significantly different. The distribution curves of the pseudo group were modified with different multiplicative transformations and then normalized. PCA analysis was calculated on a pool of distribution curves containing the pseudo and the transformed data. For each transformation, the correlation coefficient (r) between PC1 and the curve that resulted from subtracting the pseudo mean curve from the transformed mean curve was calculated. In addition the significance value between the weights of the two groups was calculated both for PC1 and PC2 (PC1, PC2). Half of the population was multiplied by a and half by b, causing more than two-fold difference between groups. A high correlation is attained even though the multiplication factors were very different. (DOCX) [file pone.0068131.s003.docx]

| **Multiplication factors(a, b)** | **2.5, 2.5** | **2.5, 6** | **2.5, 8** | **2.5, 4** |
| --- | --- | --- | --- | --- |
| **R** | 0.77 | 0.77 | 0.82 | 0.71 |
| **PC1** | ** | ** | ** | ** |
| **PC2** | ** | ** | ** | * |

**Table S2: When the difference between groups is large (>150%), a high correlation is attained even though the multiplication factors are significantly different.**

The distribution curves of the pseudo group were modified with different multiplicative transformations and then normalized. PCA analysis was calculated on a pool of distribution curves containing the pseudo and the transformed data. For each transformation, the correlation coefficient (**r**) between PC1 and the curve that resulted from subtracting the pseudo mean curve from the transformed mean curve was calculated. In addition the significance value between the weights of the two groups was calculated both for PC1 and PC2 (**PC1, PC2**).

Half of the population was multiplied by **a** and half by **b**, causing more than two-fold difference between groups. A high correlation is attained even though the multiplication factors were very different.
