# Supplementary material for: A Novel Whole-Cell Mechanism for Long-Term Memory Enhancement
Source: PLoS One. 2013 Jul 11;8(7):e68131. doi: 10.1371/journal.pone.0068131 (PMC3708920; doi:10.1371/journal.pone.0068131)
Supplement: Table S3 — When both additive and multiplicative processes underlie the difference between groups, a good correlation is attained only if one of the processes is very minor. The distribution curves of the pseudo group were modified with different transformations and then normalized. PCA analysis was calculated on a pool of distribution curves containing the pseudo and the transformed data. For each transformation, the correlation coefficient (r) between PC1 and the curve that resulted from subtracting the pseudo mean curve from the transformed mean curve was calculated. In addition the significance value between the weights of the two groups was calculated both for PC1 and PC2 (PC1, PC2). Two different processes were applied, an additive and a multiplicative process. The amplitude of 25% of the mepsc's population was increased by factor a and the amplitude of other 25% of the population was multiplied by factor b. (DOCX) [file pone.0068131.s004.docx]

| **Additive factor : a; multiplicative factor : b** | **a=0,**  **b=2.5** | **a=8, b=1.7** | **a=20**  **b=2.5** | **a=30**  **b=2.5** | **a=40**  **b=2.5** | **a=10**  **b=2.5** | **a=15**  **b=2.5** | **a=15**  **b=2** | **a=30**  **b=3** |
| --- | --- | --- | --- | --- | --- | --- | --- | --- | --- |
| **R** | 0.77 | 0.22 | 0.39 | 0.1 | -0.36 | 0.65 | 0.62 | 0.57 | 0.1 |
| **PC1** | ** | - | * | - | - | * | * | - | * |
| **PC2** | - | * | - | - | - | ** | ** | ** | - |

**Table S3: When both additive and multiplicative processes underlie the difference between groups, a good correlation is attained only if one of the processes is very minor.**

The distribution curves of the pseudo group were modified with different transformations and then normalized. PCA analysis was calculated on a pool of distribution curves containing the pseudo and the transformed data. For each transformation, the correlation coefficient (**r**) between PC1 and the curve that resulted from subtracting the pseudo mean curve from the transformed mean curve was calculated. In addition the significance value between the weights of the two groups was calculated both for PC1 and PC2 (**PC1, PC2**).

Two different processes were applied, an additive and a multiplicative process. The amplitude of 25% of the mepsc’s population was increased by factor **a** and the amplitude of other 25% of the population was multiplied by factor **b**.
